# Current Status and Future Perspectives on Machine Perfusion: A Treatment Platform to Restore and Regenerate Injured Lungs Using Cell and Cytokine Adsorption Therapy

**DOI:** 10.3390/cells11010091

**Published:** 2021-12-29

**Authors:** Anna Niroomand, Gabriel Hirdman, Franziska Olm, Sandra Lindstedt

**Affiliations:** 1 Rutgers Robert Wood Johnson Medical School, New Brunswick, NJ 08901, USA; an608@rwjms.rutgers.edu; 2 Lund Stem Cell Center, Lund University, 22242 Lund, Sweden; gabriel.hirdman@med.lu.se (G.H.); franziska.olm@med.lu.se (F.O.); 3 Wallenberg Center for Molecular Medicine, Lund University, 22242 Lund, Sweden; 4 Department of Cardiothoracic Surgery and Transplantation, Skåne University Hospital, 22242 Lund, Sweden

**Keywords:** lung transplantation, EVLP, machine perfusion, cell therapy, mesenchymal stromal cells, extracellular vesicles, cytokine adsorption

## Abstract

Since its advent in the 1990′s, ex vivo lung perfusion (EVLP) has been studied and implemented as a tool to evaluate the quality of a donor organ prior to transplantation. It provides an invaluable window of opportunity for therapeutic intervention to render marginal lungs viable for transplantation. This ultimately aligns with the need of the lung transplant field to increase the number of available donor organs given critical shortages. As transplantation is the only option for patients with end-stage lung disease, advancements in technology are needed to decrease wait-list time and mortality. This review summarizes the results from the application of EVLP as a therapeutic intervention and focuses on the use of the platform with regard to cell therapies, cell product therapies, and cytokine filtration among other technologies. This review will summarize both the clinical and translational science being conducted in these aspects and will highlight the opportunities for EVLP to be developed as a powerful tool to increase the donor lung supply.

## 1. Introduction

For patients faced with end-stage lung disease, lung transplantation is the choice of treatment for irreversible pathologies. Despite several advancements in the field, the scarcity of donor grafts and high rates of post-operative morbidities remain formidable obstacles. Disease processes, including ischemia reperfusion injury (IRI) and its most severe form of primary graft dysfunction (PGD), are common sequalae in the first 72 h following transplantation, with impairment on a clinical spectrum from mild hypoxemia to respiratory distress [1].

To address the damages inherent to the process of transplantation from the point of organ retrieval to lung reperfusion, both novel technologies and innovative treatments have been sought after.

Machine perfusion of the lungs—often referred to as ex vivo lung perfusion (EVLP)—provides a platform to answer that call. As an extracorporeal device in which harvested lungs are supported through ventilation and perfusion, the system presents an opportunity to both assess and treat the graft.

**Figure 1:**
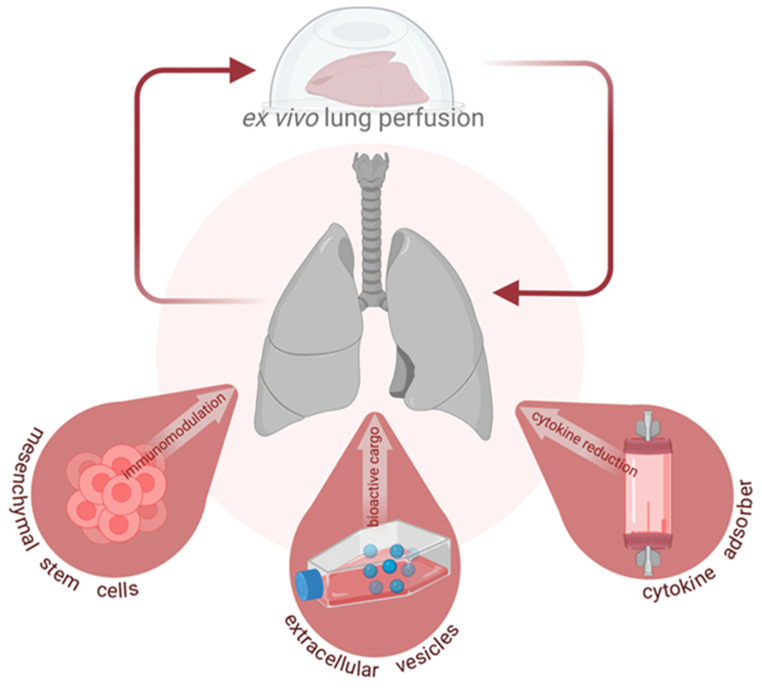
Emerging interventions during ex vivo lung perfusion (EVLP).

Developed by the Lund group, the first marginally viable donor lungs were transplanted in 2005 after reconditioning at Lund University [2,3,4,5]. A stable EVLP model operating over several hours was further established by the Toronto group and provided the basis for the evaluation of a sub-optimal lung [6,7]. Once acquired from the donor, lungs which are placed into the EVLP system can be surveilled for their quality. The use of this system for monitoring also provides a window of opportunity for the administration of targeted interventions which could recondition lungs damaged by IRI or impaired by acute lung injury (ALI). Thus, the EVLP system can be more than a means of assessing viability: rather than only surveilling lungs, proactive treatment could be applied to ameliorate damage. Treatments given during EVLP benefit from a direct and isolated connection to the lung, sparing difficulties which could arise from exposure to the systemic circulation and processes of a recipient. Several potential therapies are being tested within this system, including the use of specific molecular agents as well as placement of dialysis in line with the system [8,9,10]. This review, as a result, focuses on the use of EVLP for intervention and summarizes the studies conducted on three promising therapies: mesenchymal stromal cells (MSCs), extracellular vesicles (EVs), and cytokine adsorption, as summarized in Figure 1. These three therapies have been selected for review given their opportunity to potentiate clinical change and for the proximity of the treatments to implementation in the clinic.

Machine perfusion has served as a basis for treatment in transplantation research in other organs, especially due to the commercial ability of such devices. Systems are based off the same premises as EVLP: flow through the organ circulates preservative solution and facilitates both the delivery of oxygen and nutrients while carrying out waste and toxins. Within such circuits, kidneys and livers have been treated with drugs, including anti-inflammatory molecules, vasodilatory agents, and an array of other active compounds [11]. To call out MSC therapy in particular, models of acute renal failure and liver damage have demonstrated improved organ function following cell administration [12,13,14,15,16]. The therapy is further being explored in these organ systems for the potential of lower rates of rejection [11,13,14,16]. Extracellular vesicles given to kidneys have similarly resulted in reduced ischemic injury [17]. Given the success of these treatment applications to the kidney and liver, it is logical that such therapy can and should be explored in the lungs.

## 2. Mesenchymal Stromal (Stem) Cells

In the pursuit of methods to mediate damage from IRI and to repair impairment caused in acute lung injury (ALI), cell therapy has raised interest for a number of compelling reasons. The administration of multipotent cells, such as mesenchymal stromal (stem) cells (MSCs), has been shown to act on many inflammatory pathways and in an immunomodulatory fashion. This has a relative advantage over other therapeutic interventions which hone in on a single target, an approach which may not suffice in tackling complicated, multifactorial disease processes. MSCs have been extensively studied for their tissue regenerative properties exerted mainly through paracrine effects. There are studies showing how MSCs affect a consortium of cytokine levels. One such claim would be how MSCs are correlated with decreased levels of IL-8, a cytokine known to be released during reperfusion and correlated with graft function in lung transplantation [18]. Bone marrow-derived stromal cells have also been shown to increase IL-10 production, an anti-inflammatory cytokine [19]. The direct secretion of growth factors by the cells could be protective against ALI and part of the mechanism behind their immunomodulatory capacity [20].

In the face of any number of purported benefits, however, a given therapy must also be shown to be safe and non-harmful. The allure of MSC therapy lies in its lack of immunogenicity. This is attributed to the absence of co-stimulator molecules CD40, CD40L, CD80, and CD86 which allow the MSCs to avoid activation of effector T cells [21]. Immunogenicity is an important consideration due to the rates of both acute and chronic graft rejection mediated by the immune system which jeopardizes outcomes in lung transplantation. Any viable therapy would have to ensure that the immune system was not increasingly activated. To this end, the safety of these types of cells for human intervention at large has already been established. MSCs to treat acute respiratory distress syndrome (ARDS) are proven to be safe, with the SafeCell systematic review as well as the START trial showing no evidence of safety concerns [22,23]. In the prospective randomized START trial of one dose of 10 × 10^6^ cells/kg predicted body weight, none of the 60 treated patients across five centers experienced hemodynamic or respiratory adverse effects [22]. Mortality as well did not differ between the treatment and placebo groups. SafeCell followed prospective clinical trials who gave MSCs intravascularly and looked at 1012 participants across 26 studies who suffered from a number of clinical conditions [23]. The meta-analysis found no correlation with acute infusional toxicity, organ complications, infection, death or malignancy, which led to the determination that MSC therapy was safe with the caveat of a need for larger scaled trials. In the phase 1 REALIST trial from 2021, a specific population of CD-362-enriched MSCs were again found to be well tolerated in moderate to severe ARDS, with doses of up to 400 × 10^6^ cells per patient [24].

Once safety, viability, and efficacy are established, the hurdle of therapy manufacture must then be conquered. An advantage of cell therapy in this regard would be the ability to isolate and expand mesenchymal stromal cells from a variety of origins. The literature shows the breadth of origins from which cells can be derived, including bone marrow, adipose tissue, umbilical cords, and amniotic fluid.

Based off the successes in using MSCs to treat several disease processes, it has become increasingly popular to turn towards their potential utility within the field of lung transplantation, particularly as EVLP provides a platform through which to deliver the product directly to the target organ in a controlled manner. In early adopters of the application of MSCs to ex vivo lungs, a series of articles out of the University of California, San Francisco pointed to the potential efficacy of the treatment. In a 2009 application of bone marrow-derived MSCs or medium conditioned by MSCs during lung perfusion, human lobes damaged by intrabronchial instillation of endotoxin were found to have restored lung endothelial permeability when treated relative to untreated damaged lobes [25]. The message of MSCs ameliorating alveolar fluid clearance was emphasized by noting restored clearance after both cell or conditioned medium treatment. Keratinocyte growth factor (KGF) was reported in the study as a critical secreted factor, which is a growth factor previously tied to the reduction of lung injury within small animal models of pulmonary edema [26,27,28]. These results were followed up in 2014 by the group’s study of human lungs rejected for transplantation with decreased alveolar fluid clearance [29]. After instilling 5 × 10^6^ bone marrow-derived cells into the perfusate of a lobe one and a half hours into EVLP, the alveolar fluid clearance rate was found to be increased when re-examined 4 h later compared to baseline. The group’s extension of the endotoxin acute lung injury model (ALI) to mice in 2015 explored how bone marrow-derived MSCs given intratracheally 4 h after injury affected survival [30]. There was an improvement after 48 h, which was in part attributed to lipoxin A4 along with decreased histological evidence of injury.

Others have explored the role that MSCs could play within models of prolonged ischemia. A 2016 study from the Toronto program examined prolonged (18 h) cold ischemia, administering MSCs isolated from human umbilical cords after the first hour of EVLP [31]. The comparison of intrabronchial to intravascular instillations was combined with an evaluation of three differing intravascular concentrations. As human DNA was observed within intravascularly but not intrabronchially treated biopsies, and because airway pressures increased with intrabronchial doses, intravascular delivery was preferred. Additionally, the comparison of doses of 50 × 10^6^ cells, 150 × 10^6^, or 300 × 10^6^ cells revealed that while the middle dose of 150 × 10^6^ was an improvement over effects found in the lowest dose, the highest 300 × 10^6^ dose was not. Between the highest and middle doses, there was no physiologic advantage shown in the PaO_2_/FiO_2_ ratio or compliance. Thus 150 × 10^6^, which equates to 5 × 10^6^ MSCs per kg in these pigs, was determined to be the optimal dose, which set the “per kg” dose precedent for future studies by the group. Interestingly, in contrast with Lee et al., KGF was not increased relative to baseline within this model; rather, higher levels of vascular endothelial growth factor (VEGF) were reported in the 150 × 10^6^ dose [25,31]. Capitalizing upon these conclusions, a later publication from this group applied the same cell dose to a more prolonged condition of 24 h of cold preservation followed by 12 h of EVLP [32]. This model saw the lungs transplanted into a recipient after EVLP and followed for 4 h. These same human umbilical cord-derived MSCs showed a benefit of decreased apoptosis, supplemented with the suggestion of reduced post-transplant pulmonary edema.

Human umbilical-derived MSCs were also used by Pacienza et al. in a rat model of 2 h of warm ischemia, wherein cell treatment was analyzed for decreased histological changes following intravascular administration during EVLP [33]. Decreased neutrophil detection and increased responses to ROS damage led to the authors’ suggestion that cell therapy at the time of organ removal could help preserve a donor lung. Pivoting to bone-marrow derived multipotent adult progenitor cells (MAPCs) in a different model of prolonged cold ischemia, this alternate cell therapy was considered within the setting of EVLP in human donor lungs [34]. After 4 h of EVLP, there was less overall inflammation in treated organs as assessed by histology and by a decreased number of neutrophils and eosinophils. Another study of MAPCs to treat warm ischemia instead found that porcine lungs treated at the start of 6 h of EVLP had fewer cytokines and neutrophils in BAL [35]. There were, however, no changes to physiologic measures including PVR, compliance or PaO_2_/FiO_2_ ratio compared to controls.

The MSC effect on the increase or decrease of key cytokines varies across publications. Mordant et al. reported a rise in IL-8 within their control group that did not exist in the MSC group eventually labeled as the “optimal dose” [31]. The findings of Nakajima et al. were more expansive with higher levels of HGF and IL-4, but lower levels of IL-12, IL-18 and IFN-y in the MSC-treated group [32]. In this study, however, there was no difference in IL-8 after EVLP as Mordant et al. had observed. A decrease in TNF-α was only observed following transplantation, but not EVLP. Similarly, Lee et al.’s work did not find decreased pro-inflammatory IL-8 or TNF-α [25]. Only in the study of MAPCs in EVLP was a higher level of IL-10 found in cell therapy treatment compared to vehicle control [34]. Many of the studies were limited by relatively small sample sizes, leading to the implication that continued study of the interplay between cell treatment and cytokine levels could shed light on expected changes in concentrations. To this end, Nykänen et al. undertook an effort to gain greater control over cytokine manipulation, specifically by the genetic modification of MSCs to produce increased amounts of anti-inflammatory IL-10 [36]. These cells were then given at the start of 12 h of EVLP to 5 human lungs rejected for transplantation, with the finding that this translated to higher IL-10 in the EVLP perfusate and tissue. There were not, however, differences in PVR, oxygenation capacity and compliance, or other measures of pulmonary function.

While these studies summarized in Table 1 demonstrated findings to support the continued application of cell therapy in lung transplantation, a number of key concerns are yet to be addressed. Specifics should be pinned down, including parsing apart the true pros and cons of each origin of derivation for cells used in lung transplantation specifically. Mordant et al. offer an argument that umbilical cord MSCs could be more immunomodulatory compared to adult cells due to their neonatal origin, but a rigorous comparison would need to substantiate the claim [31]. Others have refuted this point, pointing to increased proliferation with multilayering in cultures of umbilical cord-derived MSCs which does not occur in bone marrow-derived cells due to contact inhibition [37,38]. The implication of this would be the potential for malignant development, yet clinical trials have failed to replicate tumorigenic capacity [22,23,24].

Furthermore, the technicalities involved with developing a product intended for human application and clinical trial should be considered. The use of dimethyl sulfoxide (DMSO) as cryoprotectant for the storage of a cell product needs to be weighed against its toxic effects. Unpublished data referred to by Laffey and Matthay have pointed to decreased efficacy if human MSCs were not washed prior to use in a bacterial pneumonia model in sheep [39]. Additionally, clinical trials of MSCs in ARDS have removed DMSO and cell debris prior to injection [22,40]. The literature of cell therapy within EVLP inconsistently reports on this facet of cryopreservation, which would be important in assessing product practicality and establishment of protocols for continued use. To transition an experimental treatment towards a shelved product, there is also the question of what “best by” date to assign to packaged cells. Should the percentage of live to dead cells be quantified prior to instillation, particularly given how freezing and thawing may affect a living product? Few studies reported the viability of their injected cells. Even if this was incorporated into product utilization, viability is not necessarily correlated with potency, the measure for which there is no gold standard assay. Moreover, heterogeneity within protocols used for isolation, culture, and storage raise hurdles in the direct comparison of one study’s cells to another’s. Lastly, the ease of use of “off the shelf” MSCs should be compared to the potential benefits–if any–that could arise from using cells specifically derived from the donor or recipient. Custom produced cells would certainly require increased logistical organization and planning, a luxury not always afforded in the world of transplantation, but a study of what is to be gained by using a tailored product could still prove valuable.

## 3. Extracellular Vesicles

The desire to eschew the potential adverse effects of live cell treatment coupled with the growing recognition that the benefits of stem cells may lie in their secreted products has stimulated the study of extracellular vesicles. The media used to culture the cells or the extracellular vesicles isolated from the media can be applied as therapeutic products themselves. The term extracellular vesicles (EVs) refers to non-nucleated, non-replicating membranous particles released by cells which are then categorized into a number of subclassifications including exosomes, microvesicles, and apoptotic bodies based on their size or origin [41]. Due to inconsistencies within this rapidly expanding field, the International Society for Extracellular Vesicles placed guidelines by which subtypes of EVs could be more uniformly identified. The identity of the EV rests on its site of origin, with exosomes derived from endosomes while microvesicles stem from the plasma membrane. Given the difficulties of ascertaining the precise biogenesis of isolated particles, the International Society encourages alternatively grouping by other characteristics, such as dimension, density, and composition (e.g., “small” vs. “large” EVs, or “low, middle, high density” EVs) [41]. Within the articles so far published on EVs in lung injury, the trend has been to assign exosomes and microvesicles definitions based on numerical diameters. In some instances, exosomes are described as being 50–150 nm in size compared to microvesicles that are 150–1000 nm, but it must be noted that the introductions to many of these articles are riddled with varying ranges which do not play into a consistent narrative across the existing body of literature [42,43,44,45].

Regardless of the lines drawn over how size informs classification, the particles in question have piqued scientific interest given the advantages MSC-derived EVs may confer on damaged lungs over the use of whole, live cells. The vesicles have bioactive components which may mediate pathological processes due to EV immunomodulation. Like MSCs, they are non-immunogenic, but in an improvement over their cellular counterpart, the inability of EVs to divide or differentiate shields them from concerns over tumorigenicity [46,47]. As a result, their therapeutic ability has been investigated with interest. In small animal models of IRI followed by EVLP, EVs have been successfully administered with data to support their link to improved clinical parameters as well as evidence of reduced inflammation and edema [43,48]. With rats whose lungs were subjected to ischemia and reperfusion and then placed on EVLP for three hours, doses of EVs at the 2 h mark were tied to decreased pulmonary vascular resistance and pulmonary artery pressure [48]. The concentrations of nitric oxide (NO) metabolites and NO synthase were increased in the perfusate, pointing to an induced vasodilatory state that could contribute to preservation of the lung epithelial-alveolar barrier. A similar mouse model of IRI was instead pre-treated with EVs (or MSCs) one hour prior to ischemia after which EVs or MSCs were again given as supplemented perfusate during EVLP [43]. Increases in airway resistance and pulmonary artery pressure brought on by IRI were reduced with this therapeutic intervention. Furthermore, neutrophil infiltration was also tempered by the treatment. With no reported differences between the whole cell MSC treatment which was also tested and the isolated EVs, the efficacy of EVs in both time points supports their use in response to this type of damage. The question is raised, however, as to whether a pre-damage prophylactic dose is clinically practical and at which time during EVLP doses of EVs are the most effective. To translate these findings to the clinical setting, the study’s findings imply the donor would need to be treated prior to lung collection, which is problematic not only for the recovery of other donor organs, but also for the legal and clinical standards which would need to be established for such a donor treatment to be put in place. Focus would perhaps be better placed on the treatment of damaged lungs only after procurement from the donor.

Beyond the IRI model, EVs have also been explored in the setting of damage brought on by *E. coli* and its endotoxin. In rats with acute lung injury from intratracheally instilled *E. coli*, EVs from MSCs bolstered by IFN-γ showed decreased alveolar permeability by proxy of decreased alveolar protein concentrations and increased NO synthase, as well as lower TNF-α in the alveolar fluid [49]. With mice exposed to *E. coli*’s endotoxin, microvesicles given simultaneously to the insult proved to again reduce the incidence of inflammatory cells and reduced BAL protein levels [45]. This study did compare the site of delivery, with no reported differences in both intratracheal or intravenous routes. Delay of MV dosage to 12 h after injury onset, however, did show a more modest inflammatory reduction and protein decrease. While the effect of treatment timing may be intuitive, this observed phenomenon would merit increased study to help clinicians understand the point at which EV therapy would have its maximal efficacy.

To extend these findings to both human lungs as well as scenarios in which EVLP is utilized as a platform for therapeutic intervention, lungs rejected for transplantation were treated with microvesicles. In Gennai et al.’s work, MVs were given after 1 h of alveolar fluid clearance (AFC) measurement, and the lungs subsequently underwent 6 h of EVLP [44]. AFC was increased in a dose-dependent manner as two concentrations were compared, and pulmonary artery pressure and vascular resistance were decreased. Notably, there were no differences in pO_2_, pCO_2_, or TNF-α levels by the end of EVLP between the control and treatment groups. Park et al. took the human lung model a step further by instilling *E. coli* into the lower lobes before beginning 6 h of EVLP, with two different doses of MVs tested at one hour after injury onset [50]. While AFC was again increased, significantly so compared to control, there was no dose effect in this study. Neutrophil counts were lower in the treated lungs and lung protein permeabilities were reduced. There were also no changes in PaO_2_. A consideration could then emerge that if across both studies an important indication of graft viability remained unchanged with this treatment, then how utile is the treatment really?

While these studies have all taken the approach of specifically isolating the EVs from the MSC culture medium, there is also the potential for the medium to be used as a whole without further manipulation. In application of the so-called “conditioned medium”, cultured alveolar epithelial cells were put through a form of cold ischemia and then a procedure modeling EVLP with reperfusion accomplished via fresh media [51]. Those cells exposed to the conditioned medium showed lower levels of cellular damage and attenuation of the expression of inflammatory cytokines. This simplified product which undergoes fewer processing steps could be advantageous due to an increased ease in obtaining the product. Furthermore, the use of all the conditioned media eliminates the question of what type of EV is being isolated and incorporates secreted factors that MSCs are known to produce which are not packaged into vesicles. On the other hand, conditioned media is a complex product with a number of different compounds in the solution which could pose challenges when standardizing and defining the product for clinical approval.

While promising, these studies as summarized in Table 2 as a collective do not entirely establish EVs as the superior therapeutic intervention to whole cell treatment. There are alluring advantages, including the ability to eliminate concern over DMSO, which the previous section recalls as a drawback to MSC use. While EVs could be developed as an enticing alternative, there are complications which would require serious consideration. Much of the field is unstandardized and while efforts are made to come to unified definitions and protocols, progress is hindered by even basic definitions. The literature on acute lung injury provides doses based on the number of cells from which the EVs are derived, however, according to the International Society for Extracellular Vesicles, the most commonly used quantification methods of EVs include total protein amount and total particle number [41]. Should emerging studies and those in progress amend the current precedent to instead reflect the type of quantification used by other disciplines to make comparisons to publications in other fields more accessible?

Furthermore, only Varkouhi et al. calculated dosage according to recipient weight, while others relied on a fixed volume of EVs. This adds to the argument surrounding how standardization of EV dosage would be achieved. How would a dose of EVs be calculated? How much consideration do the relative amounts of bioactive components deserve? There will likely be heterogeneity within each aliquot of EVs given that the MSCs from which they were derived are themselves from heterogenous donors. The content of the EVs and concordantly their potency and efficacy would be influenced by differences between donors, which could have broad implications for how a product is manufactured over time when many donors are needed to obtain the volumes of EVs which clinical application would require. This is already an observed concern as a study by Huang et al. demonstrated that a younger donor’s EVs had greater capacity to reduce inflammatory cell infiltration and injury severity in lungs compared to an older donor [52]. This limited report on the differences between two human donors illustrates the need for larger studies on variability and its impact on variation between EV samples. The manner in which the MSCs are cultured can also alter the contents of the EVs, with known effects of culture conditions and handling [53]. This adds another dimension in complications regarding the standardization of the product.

The entire discussion thus far has only considered EVs derived from MSCs from the angle that these exogenous vesicles could be instilled into the lung using EVLP as a platform. EVs can however be produced by many cells and rather than a treatment modality, they could be a diagnostic marker when EVs from lung tissue are analyzed. Vallabhajosyula et al. consider this vantage point as EVLP provides an EV-free starting point: perfusate can be collected after lungs are hooked up to the system and newly released vesicles can be isolated from this perfusate [54]. In a study of six lungs, of which three were eventually designated for transplantation, EVs were recovered after four hours of EVLP. EV size was increased in the non-transplanted lungs and all EVs were found to have markers demonstrating pulmonary origin. The study chiefly demonstrates that EVLP can be used as a site for EV collection and highlights the potential for harvesting EVs to serve as diagnostic markers of transplant quality. If further research were able to characterize the qualities that EVs from transplantable grafts had, they could be collected at the outset of EVLP from a lung with questionable potential and used as an early indicator of transplant suitability. This does, however, take an optimistic view of the time and effort that EV isolation and characterization currently demands. As methodology and understanding of EVs advances, the potential of these released vesicles from both the lung tissue itself for diagnostics and from MSCs for therapy is promising, but the road to standardization of production and analysis is yet long.

## 4. Cytokine Adsorption

Cytokines are known mediators of important inflammatory processes which contribute substantially to disease progression found in IRI and ARDS. Both processes are notable for the damage they wreak on donor lungs, leading to the unsuitability of the grafts for further transplantation. As such, the logical conclusion has been that the removal of cytokines from the system would aid in reducing the damage inflicted upon lung tissue and would mediate the improvement of donor organs for surgery. To accomplish this feat, purification techniques have included commercial products such as cytokine adsorbers which utilize polymer beads to target middle and low molecular weight molecules [55]. Lung transplant research can capitalize on the findings already obtained in other fields, including the use of adsorbers in human orthotopic heart transplantation and in human kidney transplantation settings [56,57]. Furthermore, a number of studies, both pre-clinical and clinical trials, examined the utility of the adsorbers in cases of severe sepsis. The reports have found reduced levels of IL-6, IL-8, IL-1β and TNF-α using the adsorber [58,59,60,61]. In clinical trials on septic patients, noradrenaline doses were reduced in treated individuals and treatment was associated with decreased neutrophils, and total white blood cell counts [58,62]. This is promising given the finding that increased IL-6 and TNF-a in the plasma and bronchoalveolar lavage (BAL) fluid in sepsis correlates with decreased survival and that higher IL-6 equates to longer time spent on a ventilator [63,64,65].

Study of these adsorbers within the context of EVLP and lung transplantation have thus far fixated on the injury that results from IRI and prolonged EVLP. In a porcine study undertaken by Kakishita et al., lungs placed on EVLP were added in line to an adsorption column which selectively adsorbed β2-microgloublin along with proinflammatory cytokines, including IL-8, IL-1β, IL-6 and TNF-α [66]. Admittedly, the degree to which TNF-α was known to be adsorbed by the column was markedly lower at 31.2% adsorption compared to the other cytokines, which ranged from 99.9% to 82.9%. With prolonged study of EVLP at 12 h of duration, the cytokine levels in the perfusate were significantly lower for TNF-α and IL-8 between treated and non-treated groups. With no significant differences measured across a number of clinical variables, including oxygenation ratio, pulmonary vascular resistance (PVR), and peak airway pressure, the clinical benefits of this particular membrane are difficult to conclude on. Given the low-to-moderate degree of injury inflicted on the tested lungs, as evidenced by the minimal histological changes seen in biopsies, the limited conclusion which can be drawn is that EVLP may feasibly be extended beyond conventional runtimes.

A more pointed exam of greater lung injury comes from two studies from Iskender et al. utilizing an IRI porcine model where lungs were kept in cold ischemia for 24 h to then be placed on 12 h of EVLP [67,68]. In their 2017 publication where a cytokine adsorber was placed in line with EVLP, the control group was notable for worsening consolidation on x-ray and the treated group benefited from improved airway pressures, dynamic compliance, and pulmonary edema [68]. The use of prolonged EVLP and the improved outcomes in the treatment group underlined the conclusions reached by Kakishita et al. These lungs with this particular adsorber, a different commercial product than Kakishita et al., were remarked to have reduced lactate levels as well as a range of diminished cytokines, including IL-1β, IL-6 and TNF-α. The 2021 publication generated from the same group expanded on these findings by undergoing the same protocol of IRI and EVLP, except that EVLP was reduced to 6 h and the left lung was transplanted into a recipient then monitored for 4 subsequent hours [67]. The treatment group was again found to similarly have reduced cytokine concentrations in the EVLP perfusate. With a shortened EVLP period, the dynamic compliance and PVR were no longer significantly different but decreased lactate levels during EVLP were a constant finding. IL-1ra, IL-6 and IL-8 were reduced after transplantation with higher dynamic compliance. While the authors highlight a lower PaO_2_/FiO_2_ ratio in the untreated group, the difference with the treated group was not statistically significant.

When contextualizing the conclusions drawn within these studies (summarized in Table 3), the findings make an argument for increased donor availability in situations in which greater time between harvest and transplant are necessary. They do not, however, necessarily advocate for the ability of the adsorption techniques to be used in lungs already damaged while in the donor. In all three studies, the grafts were healthy at the time of acquisition. The reports do continue to build a foundation for further study of cytokine adsorption, which holds particular interest given the degree to which the intervention is non-invasive. Cytokine adsorption does not depend on the infiltration of any compound or material into the parenchyma of the graft. Instead, the perfusate that flows through EVLP can simply run through the adsorber, posing a potentially reduced threat to the integrity of the delicate transplant organ. Questions linger about the effect the adsorber has on the removal of desired substances. In Iskender et al.’s publication, as an example, levels of the antibiotic meropenem were reduced substantially relative to both baseline and the control group, as was the case with methylprednisone [67]. This raises concern about the implication adsorbers have on the maintenance of therapeutic levels of drugs. For these reasons, cytokine adsorption stands as a therapy of interest to pursue in clinical trials beyond the porcine models summarized here with the understanding that side effects of adsorption on other circulating products need to be greater understood.

## 5. Conclusions

In examining these three potential avenues of treatment—mesenchymal stromal cells, extracellular vesicles, and cytokine adsorption—the relative advantages and drawbacks of each of these methods can be weighed. Cytokine adsorbers are a comparatively non-invasive addition to the EVLP circuit but may adsorb more than what the clinician bargains for, while cells and EVs are immunomodulatory but complex products. A common denominator across these treatment modalities is the use of EVLP in securing both a time and a means by which lungs can be treated for damage or stored for extended periods until transplantation. As each of these treatment options is in the exploration stage in experimental models and there are not extensive clinical trial results established in their use in lung transplantation, the field is adequately set up for advancements in the development of all three interventions. These methods hold promise as clinicians and researchers continue to serve the patients who are still in need of greater quantities and greater quality of transplantable lungs.

## Figures and Tables

**Table 1:** Studies of MSCs in EVLP.

Author	Year	Model, Subject Number	Experimental Groups	Cell Type	Cell Characteristics	Cell Dose (Total Cells)	Lung Injury Model	EVLP Length	Treatment Levels of IL-8	Treatment Levels of IL-10	Treatment Levels of TNF-α	Pulmonary Function Outcomes
Bone marrow-derived cells												
Martens et al. [35]	2017	Pig, 6/group	MAPCs vs. perfusate (control)	MAPC	Obtained from Athersys/Regenesys (Cleveland, OH, USA) Tested qPCR and flow for negative and positive markers, tube formation assay, CFSE assay	150 × 10^6^	1.5 h warm ischemia, 1 h cold ischemia	6 h	Below detection limit for both groups in BAL	Below detection limit for both groups in BAL	Decreased in BAL	No differences in compliance, oxygenation, or PVR
Fang et al. [30]	2015	Mouse, 12/group	MSCs vs. PBS	MSC	Obtained from Institute for Regenerative Medicine at Texas A&M	5 × 10^5^	In vivo ALI with 5 mg/kg IT LPS	No EVLP	-	-	Decreased in in vivo mice and in coculture of MSCs with ATII cells	Increased 48 h survival rate
McAuley et al. [29]	2014	Human, 3–4/group	MSC vs. perfusate (control)	MSC	Obtained from GMP facility at University of Minnesota, (+) markers: CD73, CD90, CD105 (−) markers: CD14, CD19, CD34, CD45, HLA-DR. Tested for trilineage differentiation	5 × 10^6^	31 +/− 6 h (control) 33 +/− 31 h (MSC) Cold ischemia	4 h	-	-	-	No differences in pulmonary arterial pressures, perfusate oxygenation, AFC restored
Lee et al. [20]	2013	Human, 3–4/group	MSC IV vs. MSC IB vs. normal lung fibroblasts (PromoCell, control)	MSC	Obtained from GMP facility at University of Minnesota; met criteria defined by ISCT	5 × 10^6^	<48 h ischemic time; followed by induction of ALI in EVLP either with 6 mg *E. coli* endotoxin or 10^9^ or 10^9^ CFU *E. coli* bacteria	6–10 h	Decrease after MSC instillation	In vitro increase in co-culture of MSC with monocytes	In vitro decrease in co-culture of MSC with monocytes	AFC restored
Lee et al. [25]	2009	Human, 3–6/group	MSC vs. conditioned medium vs. normal lung fibroblasts (PromoCell, control)	MSC	Obtained from NIH repository, Tulane Center for Gene Therapy; met criteria defined by ISCT	5 × 10^6^	21 +/− 13 h ischemic time; induced ALI in EVLP with 0.1 mg/kg *E. coli* endotoxin	4 h	MSC not different from injured control	MSC not different from injured control	MSC not different from injured control	AFC restored
Human umbilical cord perivascular cells												
Nykänen et al. [36]	2021	Human 4–5/group	MSCs in one lung vs. perfusate in matched pair lung	MSC modified to produce IL-10	(+) markers: CD73, CD90, CD105, CD10, CD166, CD140b, CD146, MHC I; (−) markers: CD34, CD45, MHC-II; transgene expression of FLAG tag for IL-10 transduction	40 × 10^6^	cold ischemia of 9 h (7.6–12.3) in control, 8.9 h (7.9–11.6) in MSC	12 h	MSC not different from injured control	Increased in tissue and perfusate	-	No difference in PVR, oxygenation, compliance, airway pressure
Pacienza et al. [33]	2019	Rat 8–10/group	MSCs vs. vehicle control of Krebs-Henseleit solution	MSC	Obtained from Laboratory of Gene Therapy at Universidad Austral, met ISCT guidelines, (+) markers: CD44, CD90, CD105 (−) markers: CD11b, CD34, CD45	1 × 10^6^	2 h warm ischemia, 90 min cold ischemia	1 h	-	-	-	Compliance decreased by less from baseline in MSC group
Nakajima et al. [32]	2019	Pig, 6/group	MSCs vs. perfusate (control)	MSC	Obtained from Tissue Regeneration Therapeutics, (+) marker: CD73	5 × 10^6^/kg	24 h cold ischemia	12 h	MSC not different from control in EVLP or post-transplant	-	MSC not different from control in EVLP, decreased post- transplant	Peak airway pressure reduced in EVLP, no change in oxygenation, PVR, compliance in EVLP or post-transplant
Stone et al. [41]	2017	Mouse 6–8/group	MSCs vs. EVs vs. Steen Solution vs. Krebs Henseleit buffer	MSCs	(+) markers: CD73, CD90, CD105, CD44 (−) markers: CD45, CD34, CD11b, CD19, HLA-DR Tested for trilineage differentiation	1 × 10^6^ before ischemia, 3 × 10^6^ in EVLP	In vivo 1 h hilar occlusion followed by 2 h reperfusion Or 1 h warm ischemia, 1 h cold ischemia followed by EVLP	1 h	-	Increased in in vivo model	Decreased in in vivo model	Increasing compliance, decreased PA pressure in both in vivo and EVLP models
Mordant et al. [31]	2016	Pig, 3–5/group	IB MSC vs. IV MSC vs. no cells	MSC	Obtained from Tissue Regeneration Therapeutics, (+) marker: CD73	IB: 50 × 10^6^ IV: 50 × 10^6^ 150 × 10^6^ 300 × 10^6^	18 h cold ischemia	12 h	Decreased in IV MSC	IV MSC not different from control	-	No change in PVR in IV MSC, transient increase in IB, Increased oxygenation, compliance with 150 × 106 IV dose.
La Francesca et al. [34]	2014	Human, 4	MAPC or sterile saline (control)	MAPC	(+) markers: CD49c, CD90 (−) markers: MHC class II, CD45	1 × 10^7^	8 h cold ischemia	4 h	-	No significant difference in tissue or BAL	-	Reduced injury on histology scoring, reduced neutrophils and eosinophils

AFC—alveolar fluid clearance; ALI—acute lung injury; ATII—alveolar type II cells; BAL—bronchoalveolar lavage; EVLP—ex vivo lung perfusion; IB—intrabronchial; IT—intratracheal; IV—intravenous; LPS—lipopolysaccharide from *E. coli*; MSC—mesenchymal stromal (stem) cells; MAPC—multipotent adult progenitor cells; PBS—phosphate buffered saline. International Society of Cellular Therapy (ISCT) Criteria: (+) markers: CD105, CD73, CD90; (−) markers: CD45, CD34, CD14 or CD11b, CD79α or CD19, HLA-DR; cells must show trilineage differentiation.

**Table 2:** Summary of extracellular vesicle studies.

Author	Year	Model, Subject Number	EV Type	Characteristics	Dose	Reported Size	Isolation Method	Origin of MSC	EVLP	Pulmonary Function Outcomes
Whole media or whole fraction of EVs										
Miceli et al. [51]	2021	Human cell line	Unmanipulated conditioned medium	No characterization of EVs	Each mL of collected medium was conditioned by 10^6^ cells, Media from 2 days of cell growth after second passage	Not Applicable	Centrifugation, unspecified	Amnion of human term placenta	Modification adapted for cultured A549 cells	-
Lonati et al. [48]	2019	Rat, 5/group	EVs	NanoSightfor distribution Reported using further FACS, western blot, and EM	0.5 mL aliquot with 24.56 ± 5.53 × 10^10^ EVs/mL diluted into 5 mL	Average diameter of 100 nm	Supernatant after overnight culture from 1 × 10^6^ cells that was centrifuged at 3000× *g* for 20 min and then 100,000× *g* for 120 min at 4 °C	Unspecified	3 h	Decreased TPVR, NO metabolites and peak pressure. No difference in compliance or oxygenation.
Varkouhi et al. [49]	2019	Rat, 8–18/group	EVs (from naïve or IFN-γ primed MSCs)	Flow cytometry with small particle detection modifications, TEM detection	100 × 10^6^ EVs/kg derived from 35–40 × 10^6^ MSCs	71.8 nm ± 15.7 nm (naïve) and 47.7 ± 25.2 nm (IFN-γ primed)	Centrifuged at 300× *g* and 2000× *g* for 10 min and then 100,000 g for 90 min at 4 °C	Human umbilical cord	No	Enhanced survival after *E. coli* pneunonia
Stone et al. [41]	2017	Mouse 6–8/group	EVs	Nanosight for size and concentration, imaging flow cytometry for CD90, CD44, CD73 and lipohilic dye, quantified protein and RNA content	1 × 10^6^ prior to ischemia and 3 × 10^6^ in EVLP	164 ± 10.4 nm	Supernatant from cells overnight was centrifuged at 10,000× *g* for 20 min and then 100,000× *g* for 1 h at 4 °C twice	Human Umbilical cord	1 h	Improved pulmonary compliance and pulmonary artery pressure
Microvesicles										
Park et al. [50]	2019	Human lungs, 5–9/group	Microvesicles	Nanosight, Labeled to separate from debris and did flow cytometry (CD9, CD44), SEM	1 × or 2 × 200uL, 10 uL is release of 10^6^ cells over 2 days	Mean size of 180 ± 14 nm	Conditioned medium collected after 48 h, centrifuged 3000 rpm for 20 min and then 100,000 for 1 h twice at 4 °C	Human bone marrow	6 h	Improved AFC, no significant difference in PAP, PVR, compliance, or oxygenation
Vallabhajosyula et al. [52]	2017	Human lungs, 6	Microvesicles	Nanosight fluorescence analysis (MHC I, MHC II, VE-cadherin, CD14, Flotillin-1, CD63, PECAM-1, cytochromeC, β-actin), RNA analysis of cargo, protein and western blot analysis proteomic profiling	EVs from the lung, isolated from perfusate	Median size 212 nm (195–240) and 165 nm (161–190) across groups	Perfusate first centrifuged at 500× *g* 10 min, then passed through Sepharose exclusion column and eluant was pooled and ultrafiltered (100-kDa cutoff) and ultracentrifugated 120,000× *g* for 4 h at 4 °C	Vesicles released by perfused human lung	up to 4 h	Larger vesicle size in lungs not transplanted
Gennai et al. [44]	2015	Human lungs 4–6/group	Microvesicles	TEM, protein content, Ang1 expression, western blot (CD44), PCR for Ang1	100 or 200 uL doses; (10 uL per 1 × 10^6^ cells)	50 to 200 nm	Media from 48 h was centrifuged at 300× *g* for 20 min and then 100,000× *g* for 1 h at 4 °C twice	Human bone marrow	8 h	Improved AFC, restored tracheal pressure, increased compliance relative to baseline. Reduced PAP or PVR. No significant different in oxygenation.
Zhu et al. [45]	2014	Mouse 14–20/group	Microvesicles	TEM, total protein, RT-PCR (Ang1), KGF/FGF7, CO1 & CO2)	15 and 30 uL (10 uL per 1 × 10^6^ cells)	Approx 200 nm	Media from 48 h was centrifuged at 3000 rpm for 20 min and then 100,000× *g* for 1 h at 4 °C twice	Human bone marrow	No	Increased protein permeability in primary cultures of ATII cells

AFC—alveolar fluid clearance; ATII—alveolar type II cells; EM—electron microscopy; EV—extracellular vesicle; FACS—fluorescence-activated cell sorting; NO—nitric oxide; PAP—pulmonary artery pressure; PVR—pulmonary vascular resistance; SEM—scanning electron microscopy; TPVR—total pulmonary vascular resistance; TEM—transmission electron microscopy.

**Table 3:** Summary of cytokine adsorption studies.

Author	Year	Model, Subject Number	Lung Injury Model	EVLP Lenght	Cytokine Filtration Type	Treatment Levels of IL-8	Treatment Levels of TNF-a	Oxygenation	Histology
Kakishita et al. [66]	2010	Porcine 5–6/group	Not applicable	12 h	Lixelle S35	Significantly lower in treatment group	Significantly lower in treatment group	No significant differences between groups	Similar levels of edema formation between groups.
Iskender et al. [68]	2017	Porcine, 5/group	24 h cold ischemia	12 h	CytoSorb adsorber	Significantly lower plasma levels of all cytokines in treatment group during EVLP.	Significantly lower in treatment group	Not studied	Significantly lower lung injury scores in treatement group.
Iskender et al. [67]	2021	Porcine, 5/group	24 h cold ischemia	6 h	CytoSorb adsorber	Significantly lower plasma levels of all cytokines in treatment group after 6 h of EVLP, however no differences found at 8 h post transplantation.	Not studied	Significantly better venoareterial oxygen pressure gradient in adsorption group after 6 h of EVLP as well as post transplantation.	Comparable microscopic lung injury scoring between the groups.

## Data Availability

No new data were created or analyzed in this study. Data sharing is not applicable.
